# Numerical Investigation of Transient Breakdown Voltage Enhancement in SOI LDMOS by Using a Step P-Type Doping Buried Layer

**DOI:** 10.3390/mi14040887

**Published:** 2023-04-20

**Authors:** Xiaoming Yang, Taiqiang Cao, Xiaohua Zhang, Tianqian Li, Hang Luo

**Affiliations:** 1 School of Electrical Engineering and Electronic Information, Xihua University, Chengdu 610039, China; 2 Department of Intelligent Systems, Hiroshima Institute of Technology, Hiroshima 7315193, Japan; 3 School of Mechanical Engineering, Sichuan University, Chengdu 610065, China

**Keywords:** silicon-on-insulator, MOS devices, deep depletion, breakdown voltage, transient breakdown voltage, step P-type doping buried layer

## Abstract

In this paper, the transient breakdown voltage (*TrBV*) of a silicon-on-insulator (SOI) laterally diffused metal-oxide-semiconductor (LDMOS) device was increased by introducing a step P-type doping buried layer (SPBL) below the buried oxide (BOX). Device simulation software MEDICI 0.13.2 was used to investigate the electrical characteristics of the new devices. When the device was turned off, the SPBL could enhance the reduced surface field (RESURF) effect and modulate the lateral electric field in the drift region to ensure that the surface electric field was evenly distributed, thus increasing the lateral breakdown voltage (*BV*_lat_). The enhancement of the RESURF effect while maintaining a high doping concentration in the drift region (*N*_d_) in the SPBL SOI LDMOS resulted in a reduction in the substrate doping concentration (*P*_sub_) and an expansion of the substrate depletion layer. Therefore, the SPBL both improved the vertical breakdown voltage (*BV*_ver_) and suppressed an increase in the specific on-resistance (*R*_on,sp_). The results of simulations showed a 14.46% higher *TrBV* and a 46.25% lower *R*_on,sp_ for the SPBL SOI LDMOS compared to those of the SOI LDMOS. As the SPBL optimized the vertical electric field at the drain, the turn-off non-breakdown time (*T*_nonbv_) of the SPBL SOI LDMOS was 65.64% longer than that of the SOI LDMOS. The SPBL SOI LDMOS also demonstrated that *TrBV* was 10% higher, *R*_on,sp_ was 37.74% lower, and *T*_nonbv_ was 10% longer than those of the double RESURF SOI LDMOS.

## 1. Introduction

In the passing years, as the number of electric vehicles, charging piles, and intelligent electronic equipment is on the rise, there is a sustained and strong demand for power MOSFET. Despite those wide bandgap semiconductor power devices such as SiC and GaN developing in leaps and bounds, silicon devices still occupy the largest market share due to their low cost and mature technology. A silicon-on-insulator (SOI) laterally diffused metal-oxide-semiconductor (LDMOS) devices offer the advantages of high speed, low loss, and easy integration and are widely used in power integrated circuits [1,2,3,4,5]. Breakdown voltage (*BV*) is an important performance indicator of the SOI LDMOS and comprises the static *BV* (*StBV*) and the transient *BV* (*TrBV*). An electron inversion layer is formed under the buried oxide (BOX) of the SOI LDMOS in static conditions, such that there is no deep depletion (DD) effect in the substrate, which sustains very little *StBV* [6,7]. Thus, the device has low *StBV*. Scholars have obtained many results after long-term research on *StBV*. Some of these results have been obtained using an analytical model of *StBV* [8,9,10,11,12,13,14], and others are related to new structures [15,16,17,18,19,20,21,22,23,24,25,26,27], in some of which *StBV* can reach more than 1000 V [25,26,27]. However, when a device is turned off rapidly, there is insufficient time for an electron inversion layer to form under the BOX, which can induce a DD effect in the substrate. The depletion layer in the substrate can sustain a portion of *TrBV* [6]. E. Napoli proposed a one-dimensional *TrBV* analytical model [7] and performed simulations and experiments [6,28,29] that verified that an appropriate reduction in the substrate doping concentration (*P*_sub_) of the SOI LDMOS can increase *TrBV*. In [30], a new device structure is proposed that achieves a good trade-off between *TrBV* and specific on-resistance (*R*_on,sp_). Like *StBV*, *TrBV* is determined by the smaller of the lateral breakdown voltage (*BV*_lat_) and the vertical breakdown voltage (*BV*_ver_). Studies have shown that although reducing the *P*_sub_ can promote expansion of the depletion layer and increase *BV*_ver_, the surface field (RESURF) effect is reduced [31]. An extremely low *P*_sub_ can lead to an increase in the surface electric field at the source and premature breakdown, thus reducing *BV*_lat_ and resulting in a substantial decrease in *TrBV* [31]. Therefore, the doping concentration in the drift region (*N*_d_) needs to be reduced simultaneously to obtain high *TrBV* [31], whereby *R*_on,sp_ is also considerably increased. Double RESURF, step doping in the drift region, and linear variable doping in the drift region techniques are commonly applied to improve *BV*_lat_ of SOI LDMOS devices [32,33,34,35]. If *BV*_lat_ limits *TrBV*, these three techniques can also enhance *TrBV*. SOI LDMOS with a P-top layer is called a double RESURF SOI LDMOS (D-RESURF SOI LDMOS), whose P-top layer can improve *BV*_lat_ and lower *R*_on,sp_ [33,34]. The lateral electric field distribution in the drift region of devices with step doping and a linear variable doping profile is more uniform than that of conventional devices. Therefore, they can achieve higher *TrBV* at the same drift region length. However, they have high *R*_on,sp_ due to the too-low doping concentration in the drift region near the source optimizing for *TrBV*, and these two techniques are often suitable for ultra-thin SOI devices.

*TrBV* is an important performance indicator for high-speed SOI LDMOS switching devices. To improve *TrBV* and suppress the increase in *R*_on,sp_, a SPBL was introduced below the BOX of the SOI LDMOS. The SPBL could optimize the lateral and vertical electric fields of the device and improve the *TrBV* of the device without increasing the *R*_on,sp_. The simulation results showed that the *TrBV* of SPBL SOI LDMOS was higher than that of SOI LDMOS and D-RESURF SOI LDMOS, and the *R*_on,sp_ was lower. As the vertical electric field was optimized, the turn-off non-breakdown time (*T*_nonbv_) of the SPBL SOI LDMOS was longer than that of the SOI LDMOS and D-RESURF SOI LDMOS.

## 2. Device Structure and Simulation Settings

**Figure 1:**
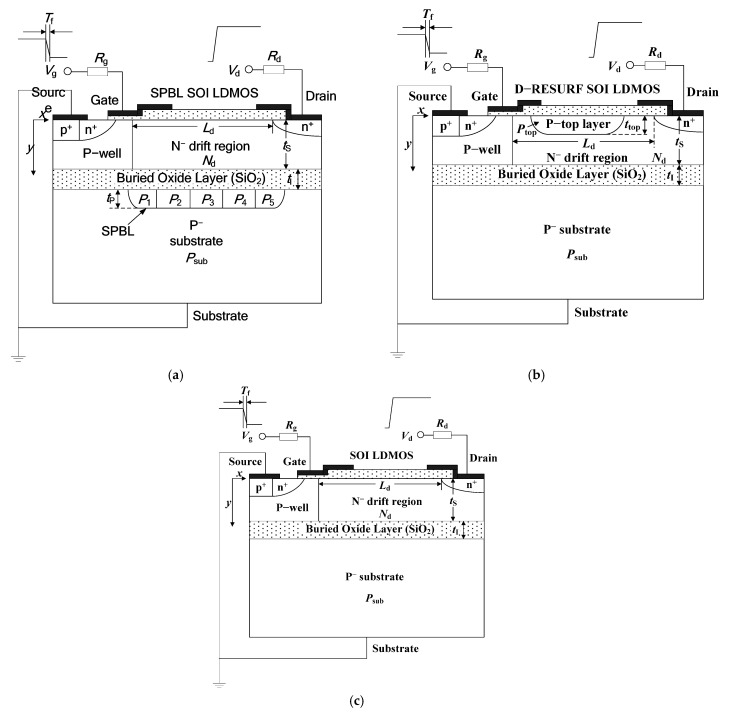
Device structure and simulation circuit of (**a**) SPBL SOI LDMOS, (**b**) D−RESURF SOI LDMOS, and (**c**) SOI LDMOS.

Figure 1a shows the SPBL SOI LDMOS device structure and simulation circuit, which differ from those of the conventional SOI LDMOS device shown in Figure 1c in that there was an SPBL below the BOX. The SPBL was divided into five-step P-type doping regions, and the doping concentration decreased from *P*_1_ to *P*_5_ by the same difference. x and y represent lateral distance from the left edge of the device and vertical distance from the top silicon surface, respectively. When the device is turned off, the high-concentration doping region of the SPBL near the source can enhance the RESURF effect and reduce the surface electric field at the source, whereas the low-concentration doping region near the drain promotes the downward expansion of the substrate depletion layer. After the SPBL is depleted, the negative charges in the SPBL exhibit a stepped distribution. This charge distribution has a significant modulation effect on the lateral electric field in the drift region, which causes the surface electric field in the middle to rise. The enhancement of the RESURF effect by the SPBL can decrease *P*_sub_. Therefore, the SPBL enables the device to maintain a small *R*_on,sp_ and increases *BV*_lat_ and *BV*_ver_. Figure 1b shows the D-RESURF SOI LDMOS. A P-type doping layer, namely the P-top layer, is on the top of the drift region. *P*_top_ and *t*_top_ are the doping concentration and depth of the P-top layer, respectively. At high *N*_d_, the source surface electric field of SOI LDMOS increases rapidly with the increase of drain voltage. However, the P-top layer in D-RESURF SOI LDMOS can effectively reduce the increasing speed of the surface electric field at the source and obtain higher *StBV*. If optimized for *StBV*, the *N*_d_ of the D-RESURF SOI LDMOS is twice that of SOI LDMOS. In other words, the *R*_on,sp_ of the D-RESURF SOI LDMOS is smaller than that of SOI LDMOS at the same *StBV*. For *TrBV*, the P-top layer can also enhance the RESURF effect, maintain high *BV*, and obtain lower *R*_on,sp_. As mentioned in the introduction, most step doping SOI LDMOS and linear variable doping SOI LDMOS are usually ultra-thin SOI devices, and their *R*_on,sp_ is high. Therefore, they were not added to the paper as a reference.

**Figure 2:**
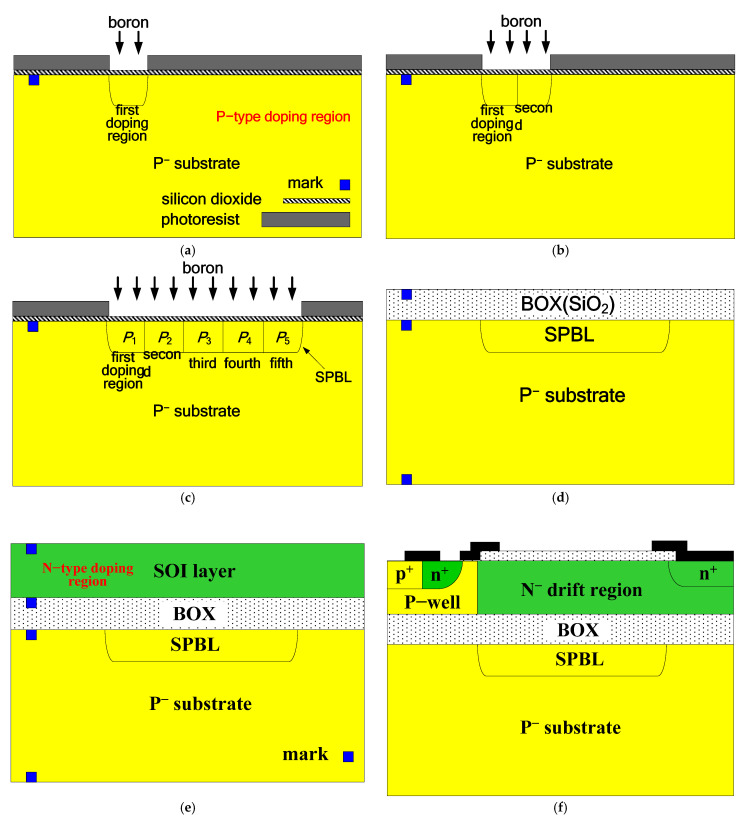
The main manufacturing process of an SPBL SOI LDMOS: (**a**) lithography alignment with marks, boron−ion implantation from the window to form the first doping region; (**b**) double the width of the window to the right, another boron−ion implantation to form the second doping region; (**c**) after five consecutive ion implantations, the formation of a SPBL; (**d**) deposition and planarization of SiO_2_ and creation of alignment marks for double−sided lithography; and (**e**) bonding of SiO_2_ and Si, thinning of the SOI layer and lithography alignment with marks. (**f**) The other manufacturing steps for the device are compatible with the standard manufacturing process for an SOI LDMOS.

Step doping profile can be achieved by adjusting the implantation window width and multiple ion implantations [34]. The main manufacturing process of the SPBL SOI LDMOS is shown in Figure 2. First, a P-type substrate was lithographically aligned, followed by boron-ion implantation from the window to form the first doping region. Next, the window width was doubled to the right, and another boron-ion implantation was performed to form the second doped region. After five consecutive ion implantations, a SPBL was formed, as in Figure 2c. Then, SiO_2_ was deposited on the P-type substrate to form a BOX; the BOX and substrate were treated with double-sided lithography for alignment marks, and the SiO_2_ was planarized. Finally, the n-type SOI layer was bonded to the BOX, the SOI layer was thinned, and the lithography was aligned with the marks. The rest of the SPBL SOI LDMOS manufacturing steps are compatible with the standard manufacturing process of the SOI LDMOS. Device fabrication steps and costs increase with the number of doping regions. Therefore, the number was two or three, and the cost could be effectively controlled.

The device parameters of the SOI LDMOS and the D-RESURF SOI LDMOS are listed in Table 1, while those of the D-RESURF SOI LDMOS are described later. To make a fair comparison of the *TrBV*s of the three devices, their *P*_sub_ was set to 2 × 10^14^ cm^−3^, and the *N*_d_ of the SOI LDMOS was optimized to 2.4 × 10^15^ cm^−3^ based on the *TrBV*. To obtain a small *R*_on,sp_ in the SPBL SOI LDMOS and the D-RESURF SOI LDMOS, *N*_d_ was set to 5 × 10^15^ cm^−3^. Optimized for *TrBV* and *R*_on,sp_, *P*_top_ and *t*_top_ in the D-RESURF SOI LDMOS were 1.1 × 10^16^ cm^−3^ and 1 μm, respectively. The other parameters of this device were the same as in SOI LDMOS. Figure 1 shows the circuit used to simulate the *TrBV* of the device [31]. The source and substrate electrodes of the test device were grounded, gate voltage *V*_g_ was applied to the gate through the gate resistor *R*_g_, and a fixed drain voltage *V*_d_ was applied to the drain through the drain resistor *R*_d_. *R*_g_ was set to a small value to ensure that the test device could be quickly turned off. The function of *R*_d_ was to limit the drain current to prevent damage to the device from an excessive current. MEDICI 0.13.2 software of Synopsys was used to perform a two-dimensional simulation of *TrBV* in the device. Several models, such as recombination, impact ionization, band-gap narrowing, mobility, and lifetime, were used in the simulation [30] The temperature (*T*) of the simulated device was 300 K by default. The breakdown condition of the device was that the drain current *I*_d_ exceeded 1 × 10^−7^ A/μm in the off-state. To simulate *TrBV*, *V*_d_ was a low positive voltage at which the test device did not break down, and *V*_g_ was decreased from 15 V to 0 V within 0.1 μs to turn the test device off. As a device is quickly turned off, there is insufficient time for an electron inversion layer to form under the BOX, the DD effect is induced in the substrate, which sustains a portion of *V*_d_ [6,7,28,29]. Then, *V*_d_ is increased until the test device breaks down. The *V*_d_ at breakdown is the *TrBV* of the test device. Every time *V*_d_ was changed, a simulation was carried out. This work was done by executing a batch file. If *V*_d_ was greater than or equal to *StBV*, the device could be broken down after being in the off-state for a period of time. The time between turning the device off and breakdown is *T*_nonbv_. To simulate the *T*_nonbv_, *V*_d_ was a voltage greater than *StBV*, and *V*_g_ was decreased from 15 V to 0 V within 0.1 μs to turn the test device off. After a period of time, *I*_d_ increased rapidly and exceeded 1 × 10^−7^ A/μm in the off-state. Data of *I*_d_ with time was stored in a log file by MEDICI. *T*_nonbv_ could be obtained by subtracting the turn-off time from the breakdown time.

## 3. Results and Discussion

**Figure 3:**
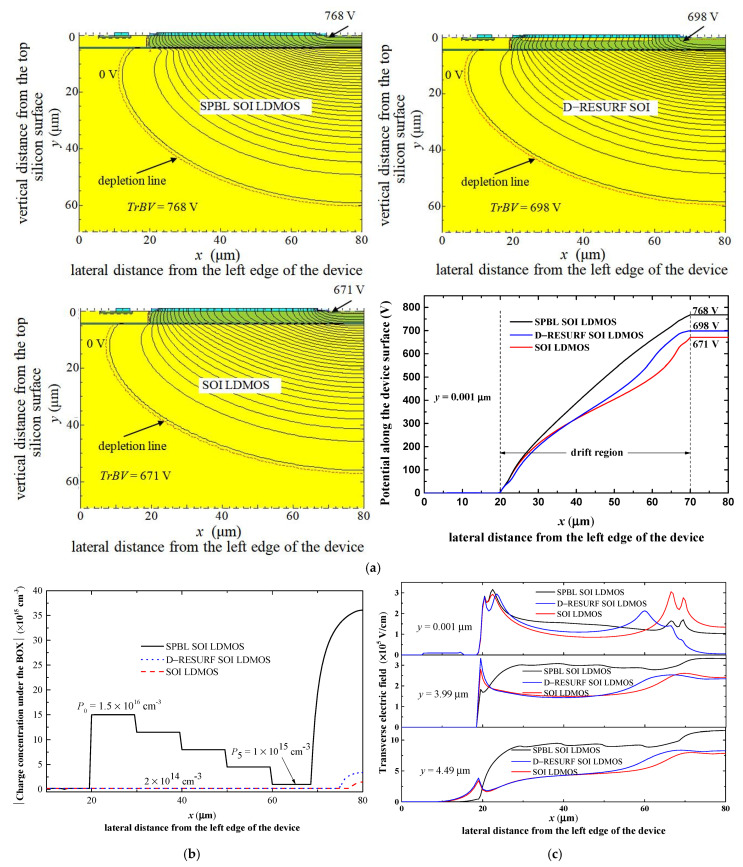
Distributions of SPBL SOI LDMOS, D−RESURF SOI LDMOS, and SOI LDMOS devices’ (**a**) potential (20 V/contour), (**b**) charge under the BOX, and (**c**) lateral electric field.

**Figure 4:**
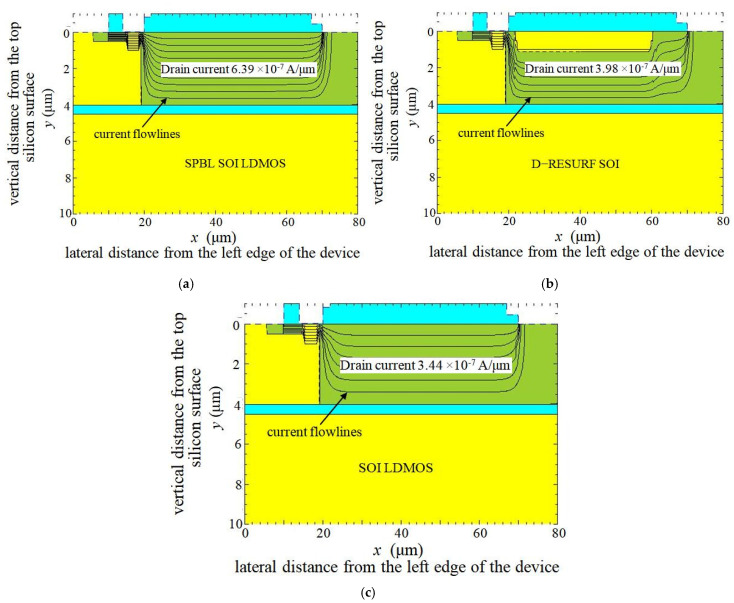
Current distributions in the drift region of (**a**) SPBL SOI LDMOS, (**b**) D−RESURF SOI LDMOS, and (**c**) SOI LDMOS at *V*_g_ = 15 V and *V*_d_ = 0.1 V.

Figure 3 shows the distribution of the charge under the BOX and lateral electric field and the potential of the three devices. The doping concentration of SPBL was reduced from *P*_1_ = 1.5 × 10^16^ cm^−3^ to *P*_5_ = 1 × 10^15^ cm^−3^ in five steps of 3.5 × 10^15^ cm^−3^ each. *TrBV* of the SPBL SOI LDMOS was 768 V, which corresponded to a 14.46% increase from that of the SOI LDMOS (671 V) and a 10% increase from that of the D-RESURF SOI LDMOS (698 V). Note that using an *N*_d_ of 5 × 10^15^ cm^−3^ for the SOI LDMOS and optimizing *P*_sub_ to 1.7 × 10^15^ cm^−3^ with respect to *TrBV* resulted in a *TrBV* of only 337 V. Figure 3a shows that compared to those of the SOI LDMOS and the D-RESURF SOI LDMOS, the distribution of equipotential lines in the drift region was more uniform and the substrate depletion layer was deeper in the SPBL SOI LDMOS, that is, the lateral electric field in the drift region was more uniform and both *BV*_lat_ and *BV*_ver_ were larger. The surface potential of SPBL SOI LDMOS in the drift region decreased from 768 V to 0 V with a relatively constant value, and the potential distribution line was relatively straight, as in Figure 3a. The decrease rate of the surface potential of the other two devices varied greatly, and the two potential distribution lines fluctuated obviously. Figure 3b shows the distribution of the charge concentration under the BOX for the three devices. There were five steps in the charge distribution below the BOX of the SPBL SOI LDMOS. The charge concentration iwas 1.5 × 10^16^ cm^−3^ in the first region near the source and decreased to 1 × 10^15^ cm^−3^ in the fifth region near the drain in fixed step sizes. These four sudden changes in the charge concentration in the SPBL could introduce spikes into the lateral electric field. The charge concentration of 2 × 10^14^ cm^−3^ under the conventional SOI LDMOS and the D-RESURF SOI LDMOS was evenly distributed, and it did not contribute to the improvement of the electric field in the middle of the drift region. Figure 3c shows the lateral electric field distribution of the three devices. Unlike with the SOI LDMOS, there were four clear electric field spikes in the lateral electric field inside the BOX (*y* = 4.49 μm) and the bottom of the drift region (*y* = 3.99 μm) of the SPBL SOI LDMOS, where the lateral electric field in the middle was higher. The surface electric field (*y* = 0.001 μm) of the new device was far from the SPBL and was weakly modulated. Although there was no electric field spike on the surface of the drift region of the SPBL SOI LDMOS, the electric field in the middle was uniform and higher than that of the conventional SOI LDMOS and the D-RESURF SOI LDMOS, indicating that the depleted SPBL modulated the lateral electric field in the drift region, which increased the *BV*_lat_ of the new device. The P-top layer of the D-RESURF SOI LDMOS could also modulate the surface electric field, but the surface electric field of the D-RESURF SOI LDMOS was not as uniform as that of the SPBL SOI LDMOS. Figure 4 shows the current distribution in the drift region of the three devices at *V*_g_ = 15 V and *V*_d_ = 0.1 V. The *N*_d_ and the current distribution area determine the drain current in the drift region. The *N*_d_ of the SPBL SOI LDMOS was 5 × 10^15^ cm^−3^, and the current distributed throughout the drift region, as in Figure 4a. Therefore, its *I*_d_ was the largest among those of the three devices. The *N*_d_ of the D-RESURF SOI LDMOS was the same as that of the SPBL SOI LDMOS, and there was no current in the P-top layer, as seen in Figure 4b. Its *I*_d_ was the second largest among those of the three devices. Although the current of the SOI LDMOS distributed throughout the drift region, the *N*_d_ was less than half that of the other two devices. Its current was the smallest among those of the three devices. According to the *I*_d_ of these three devices, their *R*_on,sp_ could be calculated. The *R*_on,sp_ was 7.82 Ω·mm^2^ for the SPBL SOI LDMOS, which was 46.25% lower than that of the SOI LDMOS (14.55 Ω·mm^2^) and 37.74% lower than that of the D-RESURF SOI LDMOS (12.56 Ω·mm^2^).

**Figure 5:**
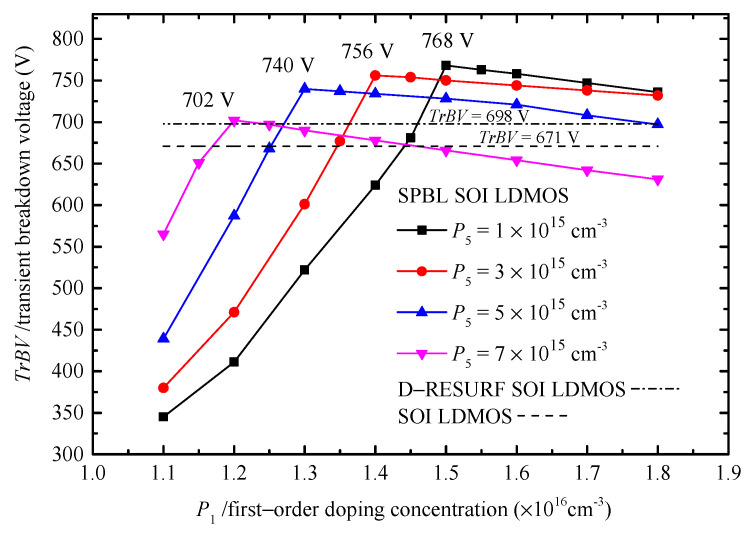
Effect of the SPBL doping concentration on the *TrBV* of the SPBL SOI LDMOS.

Figure 5 shows the effect of the SPBL doping concentration on *TrBV*. The *TrBV* of the SOI LDMOS device depended on the smaller of *BV*_lat_ and *BV*_ver_, both of which were affected by the SPBL doping concentration. Increasing the SPBL doping concentration weakened the surface electric field at the source, which was conducive to increasing *BV*_lat_ and hindered the downward expansion of the substrate depletion layer to reduce *BV*_ver_. To obtain the maximum *TrBV*, *P*_1_ and *P*_5_ needed to be optimized. At a fixed *P*_5_ and a low *P*_1_, the SPBL did not sufficiently weaken the surface electric field at the source, resulting in premature breakdown of the device at the source surface, and *BV*_lat_ was lower than *BV*_ver_. The *TrBV* was limited by *BV*_lat_. Therefore, increasing *P*_1_ could reduce the surface electric field at the source and increase *BV*_lat_, thereby increasing the *TrBV* of the device. At a *P*_5_ of 1 × 10^15^ cm^−3^, *TrBV* initially increased with *P*_1_ to a maximum value of 768 V at *P*_1_ = 1.5 × 10^16^ cm^−3^. *BV*_lat_ was equal to *BV*_ver_. As *P*_1_ increased, the doping concentration of the other regions (except for the fifth region) also increased accordingly, which enhanced the blocking effect of the SPBL on the downward expansion of the substrate depletion layer, resulting in *BV*_ver_ being lower than *BV*_lat_. The *TrBV* was limited by *BV*_ver_. Therefore, as *P*_1_ increased from 1.5 × 10^16^ cm^−3^ to 1.8 × 10^16^ cm^−3^, *TrBV* was affected by the decrease in *BV*_ver_ and continued to decrease. In short, when *P*_5_ was constant, *P*_1_ increased from low to high, *BV*_lat_ increased from low to high, and *BV*_ver_ decreased from high to low. When *BV*_lat_ was lower than *BV*_ver_, *TrBV* was determined by *BV*_lat_, and *TrBV* increased with *P*_1_. When *BV*_lat_ equaled *BV*_ver_, *TrBV* reached the maximum. When *BV*_ver_ was less than *BV*_lat_, *TrBV* was determined by *BV*_ver_, and *TrBV* decreased with the increase of *P*_1_. Similarly, increasing *P*_5_ promoted the weakening of the surface electric field at the source and increased *BV*_lat_ but hindered the downward expansion of the depletion layer and reduced *BV*_ver_. It can be seen from Figure 5 that for different *P*_5_ values, the trend of *TrBV* with increasing *P*_1_ was consistent. The larger *P*_5_, the earlier *TrBV* reached a maximum in *P*_1_. However, the increase in *P*_5_ reduced *BV*_ver_ and thereby the maximum *TrBV*.

**Figure 6:**
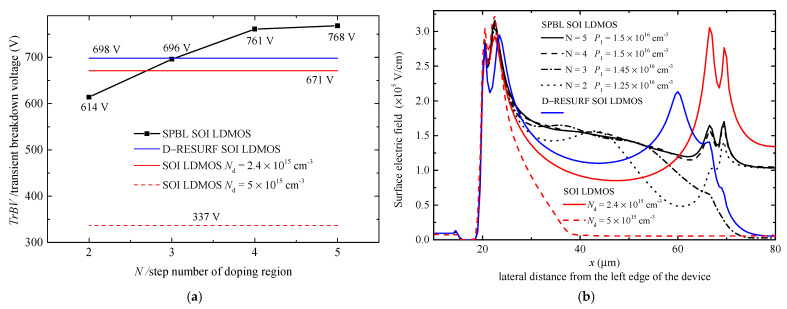
Influence of *N* on *TrBV* and surface electric field of SPBL SOI LDMOS: (**a**) the influence of *N* on *TrBV*, (**b**) influence of *N* on surface electric field distribution (*P*_N_ = 1 × 10^15^ cm^−3^).

Figure 6 shows the influence of the number of doping regions (*N*) of SPBL on *TrBV* and surface electric field. The *TrBV* of the SPBL SOI LDMOS listed in Figure 6a was the maximum value that could be gained by optimizing the doping concentration of SPBL at different *N*. The *TrBV* of the device increased with the *N* and then tended to be saturated. In Figure 6b, the surface electric fields of the SPBL SOI LDMOS were compared at different *N*. When *N* = 2, there was a comparatively high peak in the middle of the surface electric field, but the electric field near the drain was too low. *TrBV* was 614 V. When *N* = 3, there were two peaks in the middle of the surface electric field, which made the surface electric field more uniform and the electric field near the drain higher. *TrBV* increased to 696 V. When *N* = 4, the surface electric field in the middle of the drift region and near the drain was more uniform, and *TrBV* was further increased to 761 V. When *N* = 5, the surface electric field distribution was almost the same as that of *N* = 4, and *TrBV* was 768 V and only increases by 7 V. Therefore, with an increase of *N*, the SPBL modulated the surface electric field more uniformly, and a higher *TrBV* could be obtained. For the SOI LDMOS, the surface electric field at the source was too high at *N*_d_ = 5 × 10^15^ cm^−3^. The device was prematurely broken down, and its *TrBV* was 337 V. When *N*_d_ = 2.4 × 10^15^ cm^−3^, the surface electric field peaks were at the source and drain, and the electric field at the middle of the drift region was low. *TrBV* was increased to 671 V. Compared to that of the SOI LDMOS, the surface electric field of the D-RESURF SOI LDMOS in the drift region was more uniform and higher, and its *TrBV* was also 27 V higher. The *R*_on,sp_ of the SPBL SOI LDMOS was the lowest among those of the three devices. At *N* = 3, the *TrBV* of the SPBL SOI LDMOS was as high as that of the D-RESURF SOI LDMOS and higher than that of the SOI LDMOS. At *N* > 3, the *TrBV* of the SPBL SOI LDMOS was higher than that of the other two devices.

**Figure 7:**
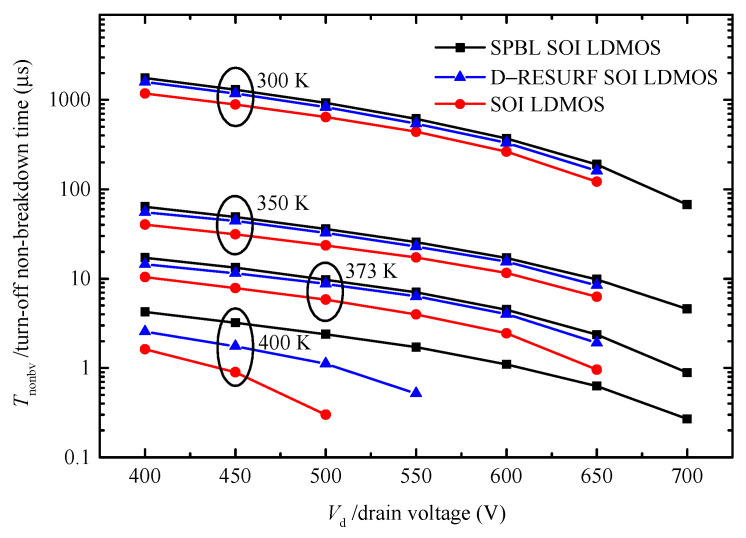
Relationship between *T*_nonbv_ and *V*_d_ at different device temperatures.

**Figure 8:**
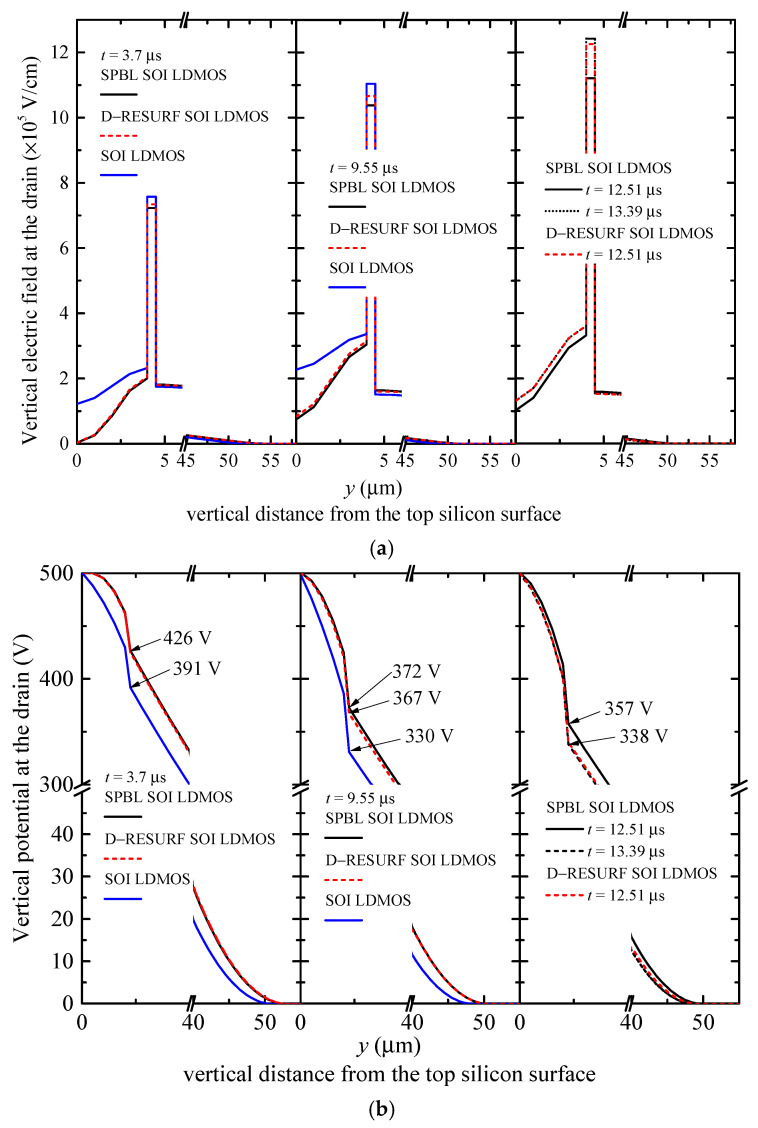
Distribution of vertical electric field and potential at the drain of the SPBL SOI LDMOS, D−RESURF SOI LDMOS and SOI LDMOS at different time points (*T* = 373 K, *V*_d_ = 500 V): (**a**) vertical electric field distribution and (**b**) vertical potential distribution.

Turning the device off generates electron-positron pairs in the substrate depletion layer. Under an electric field, the electrons move into and continuously accumulate at the bottom of the BOX, and the holes move to the edge of the depletion layer and recombine with the electrons, thereby continuously thinning the depletion layer [30]. Therefore, with increasing time, the voltage sustained by the device substrate continuously decreases, and the voltage sustained by the drift region and the BOX continuously increases. The maximum voltage that the drift region and BOX can sustain is approximately equal to *StBV*. If *V*_d_ is greater than or equal to *StBV*, the device can be broken down after being in the off-state for a period of time. *T*_nonbv_ serves as a reference for the lowest operating frequency of the device [31]. Figure 7 shows the effect of different *T*s and *V*_d_s on *T*_nonbv_. The higher the *V*_d_, the more rapid the rate of increase in the voltage sustained by the drift region and BOX, and the smaller the *T*_nonbv_. The effect of *T* on *T*_nonbv_ was more significant. The generation rate of electrons and holes in the substrate depletion layer increased rapidly with *T*, resulting in a rapid thinning of the depletion layer and a significant decrease in *T*_nonbv_. Figure 7 shows that for a constant *T*, the *T*_nonbv_ of the three devices decreased rapidly with increasing *V*_d_. The *T*_nonbv_ of the three devices decreased sharply with increasing *T*. However, for fixed *T* and *V*_d_, the SPBL SOI LDMOS always had a larger *T*_nonbv_ than the SOI LDMOS and the D-RESURF SOI LDMOS did. For a *T* of 400 K and *V*_d_ above 500 V, the substrate depletion layer of the SOI LDMOS thinned sufficiently rapidly that the device could no longer be turned off. The D-RESURF SOI LDMOS also could not be turned off when *V*_d_ was higher than 550 V. Figure 8 shows the distribution of the vertical electric field and potential at the drain of the three devices at different times for *T* = 373 K and *V*_d_ = 500 V. They were turned off at *t* = 3.7 μs: the SOI LDMOS device was broken down first at *t* = 9.55 μs, the D-RESURF SOI LDMOS device was broken down at *t* = 12.51 μs, and the SPBL SOI LDMOS was broken down at *t* = 13.39 μs. The *T*_nonbv_ of the SPBL SOI LDMOS of 9.69 μs was 65.64% longer than that of the SOI LDMOS (5.85 μs), indicating that the operating frequency had a lower minimum and a wider range for the SPBL SOI LDMOS than the SOI LDMOS. The *T*_nonbv_ of the D-RESURF SOI LDMOS was 8.81 μs, and it was slightly shorter than that of the SPBL SOI LDMOS. The vertical electric field in and the voltage sustained by the drift region and the BOX of the three devices were set to *E*_1_ and *V*_1_, respectively, and the voltage sustained by the substrate depletion layer was set to *V*_2_. Figure 8a shows that for a fixed time, the electric field at the vertical n+/n- junction at the drain of the SPBL SOI LDMOS was much lower than that of the SOI LDMOS because the *N*_d_ of the SPBL SOI LDMOS was more than twice that of the SOI LDMOS. Solving the Poisson equation with boundary conditions shows that *E*_1_ was lower for the SPBL SOI LDMOS than the SOI LDMOS. Therefore, for the same time and *V*_d_, the SPBL SOI LDMOS had a smaller *V*_1_, a larger *V*_2_, and a deeper substrate depletion layer than those of the SOI LDMOS. During device breakdown of the SPBL SOI LDMOS and SOI LDMOS, the maximum voltage sustained by the depletion layer and BOX and the substrate depletion layer depth were almost the same. Thus, the depletion layer of the SPBL SOI LDMOS was deeper than that of the SOI LDMOS during turn-off, and the depletion layer depth of both devices was equal during breakdown, such that the *T*_nonbv_ of the SPBL SOI LDMOS was longer than that of the SOI LDMOS. Figure 8b shows that at *t* = 3.7 μs and *V*_d_ = 500 V, the depth of the substrate depletion layer and *V*_1_ were 52 μm and 74 V, respectively, for the SPBL SOI LDMOS. The depth of the substrate depletion layer and *V*_1_ were 50 μm and 109 V, respectively, for the SOI LDMOS. At *t* = 9.55 μs, the depth of the substrate depletion layer of the SPBL SOI LDMOS decreased to 49.5 μm, *V*_1_ increased to 128 V, and the device was not broken down, whereas for the SOI LDMOS, the depth of the substrate depletion layer was 47.5 μm and *V*_1_ increased to 170 V, which exceeded *StBV*, resulting in device breakdown. Note that the *StBV* of the device was higher at *T* = 373 K than that at *T* = 300 K. At *t* = 13.39 μs, the depth of the substrate depletion layer of the SPBL SOI LDMOS decreased further to 48 μm, *V*_1_ increased to 162 V, and the device was broken down. Because the *N*_d_ of the D-RESURF SOI LDMOS was the same as that of the SPBL SOI LDMOS, their electric field and potential distribution were almost the same during turn-off and breakdown. The P-top layer in the D-RESURF SOI LDMOS not only weakened the electric field of the source but also enhanced the electric field of the drain. When the substrate depletion layer decreased, the *E*_1_ of the D-RESURF SOI LDMOS increased faster than that of the SPBL SOI LDMOS. The D-RESURF SOI LDMOS was broken down earlier than the SPBL SOI LDMOS. The *T*_nonbv_ of the D-RESURF SOI LDMOS was slightly shorter than that of the SPBL SOI LDMOS but longer than that of SOI LDMOS.

## 4. Conclusions

The introduction of an SPBL into an SOI LDMOS improved the *TrBV* and suppressed the increase in *R*_on,sp_ by optimizing the lateral and vertical electric fields of the device. At *t*_S_ = 4 μm, *t*_I_ = 0.5 μm, *L*_d_ = 50 μm, and *N*_d_ = 5 × 10^15^ cm^−3^, the *TrBV* of the SPBL SOI LDMOS was 768 V, which was 127.89% higher than that of the SOI LDMOS for the same *N*_d_. For a fixed *P*_sub_ = 2 × 10^14^ cm^−3^, the *TrBV* and *R*_on,sp_ were 14.46% higher and 46.25% lower, respectively, in the SPBL SOI LDMOS than in the SOI LDMOS. At *T* = 373 K and *V*_d_ = 500 V, the *T*_nonbv_ of the SPBL SOI LDMOS was 65.64% longer than that of the SOI LDMOS. The SPBL SOI LDMOS also demonstrated that *TrBV* was 10% higher, *R*_on,sp_ was 37.74% lower, and *T*_nonbv_ was 10% longer than in the D-RESURF SOI LDMOS.

Subsequent research will focus on the manufacturing process of the new device and its application in the switching power supply circuit. The process of forming a SPBL with only one ion implantation to reduce the cost will be studied. If *V*_g_ is constant at 0 V and *V*_d_ is higher than *StBV* due to a circuit fault, the device will be broken down. A protection circuit needs to be designed to avoid device breakdown.

## Figures and Tables

**Table 1:** Device parameters.

Symbol	Device Parameters	SPBL SOI LDMOS	SOI LDMOS
*L*_d_	Length of drift region	50 μm	50 μm
*L*_chan_	Channel length	5 μm	5 μm
*t*_g_	Gate oxide thickness	50 nm	50 nm
*t*_S_	Drift region thickness	4 μm	4 μm
*t*_I_	Buried oxide thickness	0.5 μm	0.5 μm
*t*_P_	SPBL thickness	1 μm	-
*P*_well_	Doping concentration in P-well	2 × 10^17^ cm^−3^	2 × 10^17^ cm^−3^
*N*_d_	Doping concentration in N^−^ drift region	5 × 10^15^ cm^−3^	2.4 × 10^15^ cm^−3^
*P*_sub_	Doping concentration of P^−^ substrate	2 × 10^14^ cm^−3^	2 × 10^14^ cm^−3^
*P*_1_	Doping concentration in first region	1.1 × 10^16^ cm^−3^ → 1.8 × 10^16^ cm^−3^	-
*P*_5_	Doping concentration in fifth region	1 × 10^15^ cm^−3^ → 7 × 10^15^ cm^−3^	-

## Data Availability

Not applicable.
